# Microangiopathy in Rheumatic Diseases

**DOI:** 10.3390/life13020491

**Published:** 2023-02-10

**Authors:** Sevdalina Nikolova Lambova

**Affiliations:** 1 Department of Propaedeutics of Internal Diseases “Prof Dr Anton Mitov”, Faculty of Medicine, Medical University of Plovdiv, 4002 Plovdiv, Bulgaria; sevdalina_n@abv.bg; 2 Department in Rheumatology, MHAT “Sveti Mina”, 4002 Plovdiv, Bulgaria

Capillaries are part of the microcirculation, which consists of arterioles, capillaries, and venules and are the connecting link between the arterial and venous blood circulation. Although the microcirculation contains only 5% of the total blood volume, it performs key functions, including exchange of oxygen and metabolites, transport of nutrients and hormones, maintenance of the homeostasis of interstitial fluids, mediation of the functional activity of the immune system and hemostasis, and control of blood pressure. Skin microcirculation takes part in the process of thermoregulation. In cases of microvascular pathology, the tissue vitality is threatened [1,2]. The capillaries are covered by endothelial cells, whose amount in the human body is estimated to be over 720 g and the predominant part of this quantity (600 g) is the capillary endothelium [3]. The endothelium controls vascular tone via the production of vasodilators, vasoconstrictors, cytokines, and growth factors. The healthy endothelium prevents thrombus formation and takes part in the process of inflammation as well as in the healing process after traumatic damage, being a vector of angiogenesis, i.e., the formation of new blood vessels from the endothelial cells of pre-existing vessels [3,4].

Interestingly, skin capillaries in the nail fold area differ, with the unique anatomical feature of being located parallel to the skin surface. For this reason, they can be observed at capillaroscopic examination along their entire length. Moreover, morphological diagnostic changes in the nail fold capillaries could appear in the early stages of systemic rheumatic diseases with peripheral vascular syndrome. These phenomena make the capillaries in the nail fold area a “window” to the diagnosis of rheumatic diseases with peripheral vascular syndrome [5].

Raynaud’s phenomenon (RP) is a common initial symptom in connective tissue diseases (CTDs). Differential diagnosis includes a broad spectrum of rheumatic and non-rheumatic disorders. Among rheumatic diseases RP is most frequent in patients with systemic sclerosis (SSc), in whom it could be found in 95% of cases. RP is also observed in 50% of patients with undifferentiated connective tissue disease, in 10–45% of those with systemic lupus erythematosus (SLE), in 33% of cases of primary Sjögren syndrome. Its prevalence in dermatomyositis/polymyositis is lower—20%, and in rheumatoid arthritis (RA) it is 10% [6].

In association with the high frequency of RP in SSc, capillaroscopic examination also reveals characteristic morphological changes, i.e., a “scleroderma”-type capillaroscopic pattern that is diagnostic for SSc in a clinical context. It is characterized by the presence of giant capillaries, microhemorrhages, devascularization, and capillary derangement [7], and could be found in the vast majority of patients with overt SSc (70–90%) [8,9]. Analogous capillaroscopic changes, i.e., “scleroderma-like” pattern, could also be observed with varying frequency in other rheumatic diseases, such as dermatomyositis (63–89%) [10], undifferentiated connective tissue disease (in approximately half of patients with RP) [11], and overlap syndromes, as well as in SLE (2–17%) [8,12,13,14,15,16,17,18,19] and RA (14–20.9%) [13,20,21,22] without signs of overlap. In SSc there is validated staging of the “scleroderma”-type capillaroscopic changes suggested by Cutolo et al. that includes three distinct phases, i.e., “early”, “active”, and “late.” The “early” phase is characterized by the appearance of a few giant capillaries; a few microhemorrhages but with preserved capillary density and architecture. In the “active” phase there are frequent giant capillary loops and microhemorrhages associated with moderate devascularization and capillary derangement. Finally, in the “late” phase capillary loss and capillary derangement are severe and there are newly formed, neoangiogenic capillaries, while microhemorrhages are absent and giant capillaries are single or absent [23]. Validated staging for “scleroderma-like” capillaroscopic changes has not been established yet; however, it has been observed that, in some cases, “scleroderma-like” capillaroscopic findings are indistinguishable from those in SSc, while in other cases the presence of discriminating features has been suggested that are to be precisely characterized [24].

Previously, it was considered that “scleroderma-like” capillaroscopic changes in SLE are associated with anti-RNP antibodies and indicate subclinical overlap with SSc [19,25], while there is now increasing evidence that “scleroderma-like” pattern could be observed in SLE patients without features of overlap and without association with anti-RNP antibodies [12,13,14,15]. In an own study (2013), a “scleroderma-like” pattern has been observed in 13.3% of examined SLE patients without signs of overlap syndrome with SSc and without association with anti-RNP antibodies. This finding was detected in patients with symptoms of secondary RP, and in half of the cases the vasculitis of digital vessels was present [12]. These observations were also later confirmed in other patient populations (van Roon et al., 2019; frequency of 17%) [13], including in children with SLE [14,15]. Of note, an association between “scleroderma-like” microangiopathy and cutaneous digital lesions in patients with both SLE and cutaneous lupus erythematosus with digital skin involvement was reported [12,26,27]. Nail fold capillaroscopy has also been suggested to play a valuable role in the evaluation of peripheral microcirculation in RA [20,28]. Initially, it was considered that “scleroderma-like” microangiopathy could not be observed in RA [17,29]. In an own study (2012), “scleroderma-like” capillaroscopic changes have been found in 14.5% of examined RA patients and by analogy with SLE, such changes were present in cases with digital vasculitis or secondary RP but without signs of overlap syndrome [20]. Later, Rajaei et al. (2017) also confirmed the presence of “scleroderma-like” capillaroscopic pattern in RA patients (frequency of 20.9%) without the presence of overlaps [21].

Capillaroscopic examination is a key method that supports the early diagnosis of SSc and other rheumatic diseases with peripheral vascular syndrome. It is a mandatory diagnostic tool in all patients with RP together with ANA screening test and if indicated in a clinical context—a test for disease-specific antibodies. The existence of an association between microangiopathy and autoantibodies (diagnostic and pathogenic) is a question that is insufficiently studied in contemporary rheumatology. In SSc, anti-Scl70 antibody positivity is suggested to be related to the earlier appearance of more advanced microvascular changes (i.e., “late” capillaroscopic patterns) [30,31,32]. In an own study, it has been found, as a novel observation, that positive anti-RNA polymerase III-155 antibodies might be detected in SSc patients without microvascular pathology in the nail fold area or with early microangiopathy [32]. These data suggest the presence of a link between immunological and microvascular disturbances and require further research for both diagnostic and pathogenic antibodies.

Among rheumatic diseases, microangiopathy is not only the most prevalent in SSc, but also in scleroderma patients it is characterized with the greatest severity and extent. Moreover, vascular damage is suggested to be the initial pathological event of the disease that subsequently triggers inflammation and fibrosis. This notion underlines the high value of microvascular assessment in the initial stages of the disease. Endothelial injury in SSc that is initiated by an unknown agent, i.e., free oxygen radical, microbes, antibodies, chemicals, is characterized by the development of dysbalance between the levels of vasodilators (nitric oxide, prostacyclin, etc.) and vasoconstrictors (endothelin, angiotensin, etc.), with the prevalence of vasoconstrictors. Damaged endothelial cells of the microcirculation mediate the proliferation and contraction of smooth muscle cells, promote the formation of microthrombi due to the impairment of the antithrombotic function of the endothelium and lead to reduction in fibrinolysis. There is overexpression and release of adhesion molecules that trigger inflammation. These pathological processes lead to tissue hypoxia, inflammation, and subsequent fibrosis [33,34,35,36]. Disturbances in vascular recovery are also characteristic for SSc, which affect both the processes of angiogenesis (new blood vessel formation from the endothelial cells of pre-existing vessels) and vasculogenesis (formation of new vessels from the endothelial progenitor cells derived from bone marrow) [37,38,39,40].

Due to the profound endothelial damage, tissue hypoxia, defective angio- and vasculogenesis, and the accompanying fibrotic process in SSc, ulcerations with different localizations are frequent findings. Digital ulcers are among the most common type of ulcerations in SSc, which include ischemic lesions at the finger pads, digital ulcers over bony prominences, and over calcifications, digital necrotic lesions. Digital ulcers in SSc require a complex therapeutic approach that includes the administration of vasodilators, local antiseptic treatment, antiplatelet drugs, anticoagulants, and antibiotics in some cases [41]. However, due to the presence of generalized microangiopathy in SSc, non-digital ulcerations can be observed at different body areas that are less well-studied. They may develop over bony prominences, over calcinosis, and on lower extremities [42]. Their etiology is complex and includes microvascular damage at the levels of the skin, tendons, fascia [42,43], and disturbances in tissue repair, i.e., the reduced proliferative potential of mesenchymal stem cells, defective circulating endothelial progenitor cells with ineffective vasculogenesis that results in impaired wound healing, development of chronic skin ulcers [42], and compromised physical capacity [43]. Thus, the treatment of ulcerations in SSc is a significant challenge in clinical practice. Novel therapeutic strategies have been suggested, such as local administration of mesenchymal stem cells derived from bone marrow or adipose tissue and initial promising results have been reported in patients with chronic skin ulcers resistant to treatment, including in SSc cases [44,45]. In a recent paper of Biehl et al. (2022), a new approach to ulcerations of lower extremities associated with calcinosis in SSc has been presented that includes the surgical removal of calcifications, vacuum therapy for the improvement of vascularization, and tissue perfusion by stimulating neoangiogenesis, lymphatic drainage, and intensified physiotherapy [43]. Further observations of the therapeutic efficacy of these new strategies are awaited to improve the complex care of SSc patients with chronic skin ulcers resistant to treatment.

In conclusion, microvascular damage plays a key role in a number of rheumatic diseases. In SSc and CTD associated with peripheral vascular syndrome, microvascular pathology is readily assessed in the nail fold area that facilitates the early diagnosis. A better understanding of the different aspects of microvascular abnormalities in rheumatic diseases might improve the diagnosis of the early microangiopathy and expand the knowledge of disease pathogenesis.
